# Behavior of *Escherichia coli* and *Salmonella* Enteritidis in Cilantro Crops (*Coriandrum sativum* L.) and the Antimicrobial Effect of *Salvia officinalis* Essential Oil

**DOI:** 10.1155/ijfo/9053569

**Published:** 2026-08-12

**Authors:** Janeth Gasca-Corona, María L. Luna-Guevara, Jesús F. López-Olguín, Dionicio Juárez-Ramón, Ynes Ortega, Miguel A. Villalobos López, Juan J. Luna-Guevara

**Affiliations:** ^1^ Agroecology Center, Institute of Sciences, Benemérita Universidad Autónoma de Puebla, San Pedro Zacachimalpa, Puebla, Mexico, buap.mx; ^2^ College of Food Engineering, Faculty of Chemical Engineering, Benemérita Universidad Autónoma de Puebla, Col. San Manuel, Mexico, buap.mx; ^3^ Herbarium and Botanical Garden, VIEP, Benemérita Universidad Autónoma de Puebla, Col. San Manuel, Mexico, buap.mx; ^4^ College of Agricultural and Environmental Sciences, Center for Food Safety, University of Georgia Griffin Campus, Griffin, Georgia, USA; ^5^ Centro de Investigación en Biotecnología Aplicada, Laboratorio de Genómica Funcional y Biotecnología de Plantas, Instituto Politécnico Nacional, Tepetitla de Lardizábal, Tlaxcala, Mexico, ipn.mx; ^6^ College of Food Engineering, Faculty of Chemical Engineering, Benemérita Universidad Autónoma de Puebla, Col. San Manuel, Mexico, buap.mx

**Keywords:** antimicrobial effect, cilantro, *E. coli*, persistence times, *S.* Enteritidis, sage

## Abstract

Fresh, ready‐to‐eat vegetables are susceptible to contamination by several bacterial pathogens. This study evaluated the behavior of inoculated *Escherichia coli* and *Salmonella* Enteritidis (7 Log_10_ CFU/g) through viable cell counts on eosin methylene blue (EMB) and brilliant green agar (BGA) in soil, seeds, and cilantro plants. Samples were analyzed at 2 h, 3, 7, 15, and 60 days (d) (the typical harvest period) under greenhouse conditions. Confirmation of both microorganisms was achieved with a combination of biochemical and molecular analyses, including IMViC tests and 16S rDNA sequencing. The essential oil (EO) of *Salvia officinalis* (0.3 *μ*L/mL, v/v) was obtained by steam distillation and was characterized by gas chromatography–mass spectrometry. The antimicrobial effect of the EO was evaluated on inoculated soil, seeds, and cilantro plants. *E. coli* persisted at 6.5 and 5.15 Log_10_ CFU/g in soil and seeds, respectively. The highest count (9.61 Log_10_ CFU/g) was observed at 7 d. *S.* Enteritidis reached populations of 7.44 Log_10_ CFU/g in soil and 7.25 Log_10_ CFU/g in seeds at 60 d. EO application resulted in reductions of 7.5 Log_10_ CFU/g with *E. coli* and 7.59 Log_10_ CFU/g for *S.* Enteritidis on inoculated cilantro plants after 60 d. These findings emphasize the potential of *S. officinalis* EO as a natural antimicrobial agent during cilantro growth.

## 1. Introduction

Food safety is a global priority due to the increasing risk of microbial contamination in food, particularly in fresh produce. Additionally, the growing consumer demand for fresh and minimally processed foods has contributed to a rise in foodborne illnesses. Several outbreaks associated with contaminated vegetables have highlighted critical vulnerabilities in agricultural production systems [1]. Among highly demanded fresh produce is cilantro (*Coriandrum sativum* L.), a widely used ingredient in global gastronomy and especially valued in Mexican cuisine for its distinctive flavor, aroma, and nutritional benefits [2, 3]. However, leaves and stems of cilantro are highly susceptible to microbial contamination during preharvesting and postharvest handling, making them a critical situation of concern for food safety management [4]. According to Leyva‐Abascal et al. [5], the stages of cilantro production include cultivation, harvesting, and postharvest processing conditions, where maintaining product quality and ensuring compliance with safety standards are essential.

Pathogens such as *Escherichia coli* and *Salmonella Enteritidis* have been frequently identified in cilantro‐related foodborne outbreaks [6], leading to significant economic losses for producers [5]. These pathogens can colonize soil, seeds, and plant tissues, making their elimination particularly challenging in agricultural systems. Traditionally, control strategies have relied on chemical disinfectants and synthetic antimicrobials; however, the increasing resistance of several pathogens to these agents highlights the urgent need to develop novel antibacterial treatments with alternative targets or mechanisms of action.

Natural antimicrobial agents derived from plant materials or their by‐products represent an innovative and sustainable approach to controlling foodborne pathogens and human clinical infections [7]. In recent years, alternative antimicrobial strategies including ferroptosis mechanisms and nanocarrier systems have emerged as promising tools to overcome antimicrobial resistance [7, 8]. In this context, plant‐derived essential oils (EOs) have achieved increasing attention due to their broad‐spectrum antimicrobial activity and potential applications in food preservation and agricultural biocontrol [9, 10]. Likewise, the constituents of EOs have their own bioactivities and are conferred with a wide range of properties; for example, oregano and thyme both contain carvacrol and thymol, which have antimicrobial and antioxidant activity; however, the EOs differ between both plants. The major active constituents of EOs, such as terpenoids and phenolic compounds, have been shown to disrupt bacterial cell membranes, interfere with metabolic processes, and inhibit biofilm development [11].

Recent studies have also demonstrated that EOs may interfere with quorum sensing pathways, biofilm formation, membrane permeability, and oxidative stress responses in foodborne pathogens, highlighting their potential as sustainable antimicrobial agents in food and agricultural systems. Furthermore, advances in EO research have demonstrated their effectiveness against multidrug‐resistant bacteria and foodborne microorganisms, supporting their potential application in agricultural and food safety systems [12, 13]. EOs and their bioactive constituents have demonstrated the ability to disrupt microbial cell membranes, induce oxidative stress through reactive oxygen species (ROS) accumulation, interfere with ergosterol biosynthesis, and inhibit the growth of spoilage microorganisms [14, 15]. In addition, synergistic combinations of EO constituents have shown enhanced antimicrobial efficacy while reducing the concentrations required for microbial inhibition, representing a promising strategy to minimize sensory alterations in food systems [16]. Advances in nanoemulsion and encapsulation technologies have also improved the stability, bioavailability, and antimicrobial performance of plant EOs under complex environmental conditions, supporting their application as innovative alternatives to conventional chemical preservatives [17].

*Salvia officinalis* is an aromatic plant known for its antimicrobial properties, which are mainly attributed to its flavonoid and terpenoid content. These bioactive compounds have demonstrated inhibitory effects against antibiotic‐resistant gram‐negative bacteria, including *E. coli* and *S.* Enteritidis [18, 19]. Moreover, the EO of *S. officinalis* has been classified by the U.S. Food and Drug Administration (FDA) as Generally Recognized as Safe (GRAS), supporting its potential use in food‐related applications.

To date, there are no reports specifically addressing the behavior of enterobacteria in soil, seeds, and fresh cilantro during production, nor the inhibitory effects of *S. officinalis* EO throughout the cilantro production system. Therefore, this study aimed to evaluate the antimicrobial capacity of *S. officinalis* EO against *E. coli* and *S*. Enteritidis in soil, seeds, and cilantro plants.

## 2. Materials and Methods

**Figure 1:**
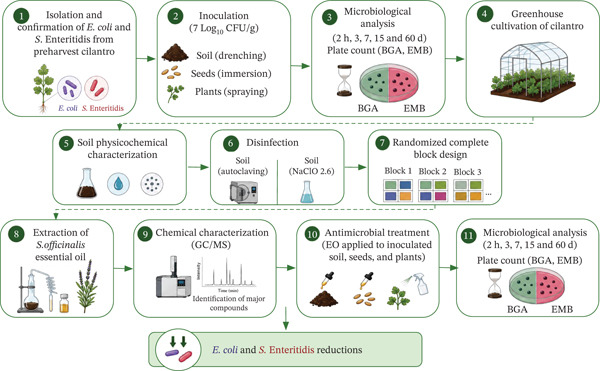
General diagram of methodology to evaluate the behavior of *Escherichia coli* and *Salmonella* Enteritidis in cilantro crops and the antimicrobial effect of *Salvia officinalis* essential oil in soil, seed, and cilantro plant, where: EMB, eosin methylene blue; BGA, brilliant green agar; EO, essential oil; and GC/MS, gas chromatography–mass spectrometry.

The following diagram illustrates the methodology used in this research (Figure 1).

### 2.1. Materials for Cilantro Growing

Agricultural soil was collected from a single site in the Central‐Eastern region of the State of Puebla, Mexico. Its physicochemical characteristics included a clay‐loam texture, pH of 8.4, and moisture content of 20.05%. Cilantro seeds (*C. sativum* L.), “Emperador” variety (commonly used by local farmers) had a moisture content of 18.06%.

### 2.2. Controlled Conditions for Cilantro Production

The experiment was conducted in a greenhouse measuring 5.89 m in length and 3.75 m in width, located at the Agroecology Center of the Benemerita Universidad Autonoma de Puebla, from July to September 2023. An EXTECH datalogger (Model 42280) was used to record temperature (T) and relative humidity (RH) every hour, and the averages were 26^°^C ± 3^°^C and 48%, respectively. These conditions closely resembled those typically found at cilantro production sites [5]. The experiment used 36 pots (disinfected with 2.6% sodium hypochlorite) and was supplemented with 7 kg of sterilized soil. The sterilized soil was autoclaved at 121°C and 15 psi for 20 min [20]. The pots were arranged in a randomized complete block design (RCBD) with four blocks, each containing all treatments (Table 1).

**Table 1:** The assignment of treatments within each block followed a randomized complete block design (RCBD) for distribution in the greenhouse.

Blocks	Treatment
I	T2, T6, T3, T1, T4, T5, T8, T9, T7
II	T5, T6, T4, T3, T2, T1, T9, T7, T8
III	T1, T4, T3, T6, T2, T5, T8, T9, T7
IV	T4, T3, T6, T1, T2, T7, T9, T8, T5

*Note:* The treatments were as follows: T1, inoculated soil; T2, uninoculated soil; T3, inoculated seeds; T4, uninoculated seeds; T5, inoculated cilantro plant; T6, uninoculated cilantro plant; T7, inoculated soil with *S. officinalis* EO; T8, inoculated seeds with *S. officinalis* EO; and T9, inoculated cilantro plant with *S. officinalis.*

### 2.3. Salvia (*S. officinalis*) EO Extraction

Sage was sourced from the region of Atlixco, Puebla, Mexico. Leaves and stems were selected and dried in an oven (Luzeren, Model RI 115‐U) at 37°C for 48 h. Briefly, 150 g of dehydrated plant material and 1500 mL of distilled water were used for the distillation process, with an extraction time of 90 min. EO extraction was performed by steam distillation using a Clevenger‐type apparatus according to Conde‐Hernández et al. [21], with slight modifications, who confirmed the extraction of antimicrobial and antioxidant compounds from *S. officinalis*. The obtained EO was collected and stored at 4°C until further use.

### 2.4. Characterization of the EO

The EO of sage was characterized by gas chromatography–mass spectrometry (GC‐MS) following the methodology described by Conde‐Hernández et al. [21]. Analyses were performed using a gas chromatograph equipped with a 5975 quadrupole mass selective detector (Agilent Technologies 6850 N GC, Santa Clara, California, United States) and an HP5‐MS column (30 m × 0.25 mm). The operating conditions were as follows: helium as carrier gas at a flow rate of 15.5 mL/min, injector temperature of 250°C, injection volume of 1 *μ*L, and split ratio of 10:1. The oven temperature program started at 60°C and increased at 4°C/min until reaching 250°C. The ionization energy was set at 70 eV, and the scan mass range was 43–350 m/z. Compound identification was performed by comparing the obtained spectra with those from the National Institute of Standards and Technology (NIST) database and relevant literature [22, 23].

### 2.5. Bacterial Strains and Inoculum Preparation

*E. coli* and *S.* Enteritidis were isolated from cilantro crops during two sampling periods, November 2021 and March 2022. Composite samples (soils and cilantro plants) were collected from five different locations of Puebla′s main cilantro‐producing regions. This study is aimed at ascertaining the persistence of bacteria during cilantro production. Samples of soil, seeds, and cilantro tissues (10 g each) were homogenized in 90 mL of sterile buffered peptone water using a stomacher for 2 min. Serial dilutions were prepared and plated on selective media: eosin methylene blue (EMB) agar equipment (Bioxon, Mexico) for presumptive identification of *E. coli*, and brilliant green agar (BGA; Bioxon, Mexico) for *Salmonella* spp. Five colonies of each sample, with typical morphology, were randomly selected and subjected to IMViC biochemical analyses. For *Salmonell*a identification, additional tests such as H_2_S production and absence of lactose fermentation were considered. Genomic DNA was extracted using the Quick‐DNA/RNA Miniprep Kit (Zymo Research, United States), and partial amplification of the 16S rRNA gene was performed using primers fD1 and rD1. PCR products were purified with the Zymoclean Gel DNA Recovery Kit (Zymo Research) and sequenced at the IBT‐UNAM Sequencing Unit. Partial sequences of the 16S rRNA gene were compared with those already existing in the GenBank of the National Center for Biotechnology Information (NCBI) to confirm their identity. To prepare the bacterial inoculum, resistance to the antibiotic nalidixic acid (NA) (Sigma‐Aldrich, Mexico) (0.5 g/mL) was induced in both bacterial strains. NA resistance was utilized as a selective marker in the context of studies of pathogen‐inoculated soil, seeds, and plants of cilantro. The protocol utilized in this study was consistent with the approach documented by Zhang et al. [24] and Shaheen et al. [25].

Fresh cultures of *E. coli* and *S.* Enteritidis were obtained from stock cultures by inoculating sterile Trypticase Soy Broth (TSB) and incubating for 18 h at 37°C. The resulting cultures exhibited a cell density of 10^8^ CFU/mL. The TSB was adjusted with sterile‐filtered (0.22 *μ*m) NA in concentrations of 0.1, 0.2, 0.3, 0.4, or 0.5 g/mL. The highest concentration of 0.5 g/mL was used for NA resistance reported by Shaheen et al. [25].

### 2.6. Soil, Seeds, and Cilantro Plants Inoculation

*E. coli* and *S.* Enteritidis were grown together in TSB supplemented with NA to attain a final concentration of 7 Log_10_ CFU/mL, the inoculum level was selected based on Markland et al. [26] that employed similar concentrations to ensure detectable pathogen populations during challenge assays. The potting soil was inoculated with 300 mL of bacterial coculture, thoroughly mixed by hand, and transferred to the greenhouse. Seeds were inoculated by immersion in the bacterial suspension (TSB + NA), and 140 seeds were subsequently sown per pot [27, 28]. Noninoculated seeds (which were disinfected by immersion in a sodium hypochlorite solution 2.6% (v/v) for 15 min) were sown in noninoculated soil to establish a control group. Following a 3‐week period, cilantro plants were inoculated by spraying them with the coculture [29]. Irrigation was carried out manually four times per day with distilled, sterilized water to maintain a field capacity (FC) of 86%.

### 2.7. Antimicrobial Treatments

An antimicrobial dispersion was prepared by mixing 3 mL of *S. officinalis* EO (0.3 *μ*L/mL, v/v) in 1 L of sterile water. The mixture was vigorously vortexed immediately before application to obtain a temporary homogeneous dispersion due to the relatively low density of the EO of 0.91–0.93 [30]. This dispersion was used for treatments applied as immersion to inoculated seeds, drenching to soil, and foliar spraying to cilantro plants, respectively. A single application of EO treatments was performed at the beginning of the experiment, with a contact time of 15 min. Specifically, inoculated soil treatments followed the protocol described by Kumar et al. [31], in which 7 kg of soil were manually homogenized with the EO dispersion (1 L) for 1 min. This method was selected to ensure complete homogenization and consistent exposure of the microbial population to the EO.

### 2.8. Microbiological Analysis

The microbiological enumeration of soil, seeds, and cilantro plants, with and without *S. officinalis* EO, was carried out at 2 h, 3, 7, 15, and 60 days (the final count corresponding to the cilantro harvest). Samples were analyzed using serial dilutions from 10^−2^ to 10^−7^ plated on EMB and BGA media with NA (0.5 g/mL) for *E. coli* and *S.* Enteritidis, respectively, incubated for 24 h at 37°C, and were performed in duplicate. After 21 days, cilantro seeds started to germinate, and the plants were analyzed [29, 32]. Presumptive identification of *E. coli* and *S.* Enteritidis was confirmed using IMViC biochemical tests [33]. In addition, a commercial rapid detection kit for *S.* Enteritidis was employed according to the manufacturer′s instructions; the kit Rapid Chek SELECT was kindly provided by PhD Silva (Mississippi State University, United States).

### 2.9. Statistical Analysis

The results of the microbiological counts of persistence and survival were obtained in quadruplicate from an independent experiment; the values were analyzed using ANOVA with *α* = 0.05 and Tukey′s test. Data processing was performed using Statgraphics Centurion software (Version 16.1).

## 3. Results

### 3.1. Characterization of *S. officinalis* EO

A comprehensive GC‐MS analysis of the EO of *S. officinalis* identified 19 compounds at higher concentrations. Of these, seven are of particular interest due to their recognized antimicrobial properties. As shown in Table 2, camphor, 1,8‐cineol, and camphene presented the highest proportion.

**Table 2:** Characterization of *S. officinalis* essential oil identified by GC‐MS.

Chemical compound	Peak area (%)
Camphor	34.17
Caryophyllene	5.58
Camphene	12.31
Borneol	3.42
*α*‐Terpineol	1.14
Sabinene	5.43
1,8 cineol	13.73

Abbreviation: GC‐MS, gas chromatography–mass spectrometry.

**Figure 2:**
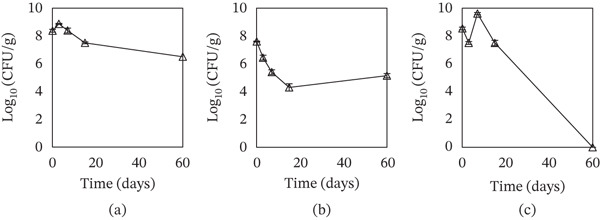
Persistence of *E. coli* in (a) soil, (b) seeds, and (c) cilantro plants.

**Figure 3:**
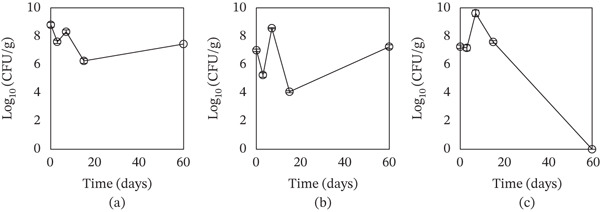
Persistence of *S.* Enteritidis in (a) soil, (b) seeds, and (c) cilantro plants.

Figure 2 considers the highest survivance of *E. coli* was observed in soil, whereas Figure 3 shows that the greatest persistence of *S.* Enteritidis observed in seeds at 60 days. Regarding *E. coli* behavior, the population was approximately 8.37 Log_10_ CFU/g at 2 h postinoculation and did not change significantly at 7 days (8.39 Log_10_ CFU/g). Finally, the population of *E. coli* in inoculated soil remained at 6.5 Log_10_ CFU/g at harvest (60 days), indicating that the bacteria persisted throughout the cilantro production (Figure 2a). *S.* Enteritidis counts increased 1.8 Log_10_ CFU/g at 2 h and persisted at 8.31 ± 0.12 Log_10_ CFU/g at 7 days, with no significant change at 7.44 Log_10_ CFU/g in inoculated soil at 60 days (Figure 3a). This persistence in both microorganisms suggests a potential microbiological hazard to fresh produce in contact with contaminated soil, particularly during harvest. While the counts of *E. coli* reached 0.6 Log_10_ CFU/g in seed after inoculation and then gradually declined, remaining at 5.15 Log_10_ CFU/g at 60 days (Figure 2b). Similarly, *S*. Enteritidis showed no change at 2 h postinoculation, increased by 2.56 Log_10_ CFU/g at 7 days, and persisted at 7.25 Log_10_ CFU/g in seeds at 60 days (Figure 3b).

The population of *E. coli* increased by 1.54 Log_10_ CFU/g at 2 h and persisted at 9.61 ± 0.07 Log_10_ CFU/g at 7 days, indicating the highest population in inoculated cilantro plants; however, bacterial counts decreased to undetectable levels by 60 days (Figure 2c). Likewise, *S.* Enteritidis persisted, with a maximum of 9.62 ± 0.23 Log_10_ CFU/g at 7 days; counts were 7.5 Log_10_ CFU/g at 15 days, and no viable cells were detected in inoculated cilantro plants at 60 days (Figure 3c).

### 3.2. Antimicrobial Effect of *S. officinalis* EO on *E. coli* and *S.* Enteritidis in Soil, Seeds, and Cilantro Plants

**Figure 4:**
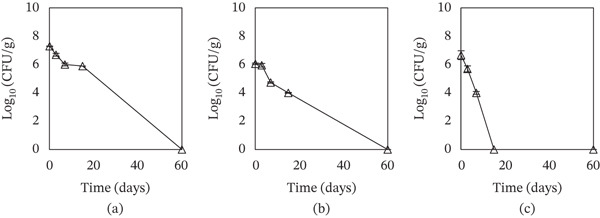
Antimicrobial effect of *S. officinalis* essential oil in *E. coli*: (a) soil, (b) seeds, and (c) cilantro plants.

**Figure 5:**
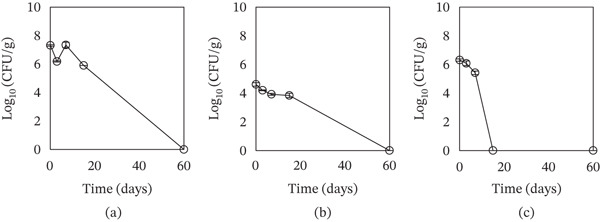
Antimicrobial effect of *S. officinalis* essential oil in *S.* Enteritidis: (a) soil, (b) seeds, and (c) cilantro plants.

The reductions were determined and compared after applying *S. officinalis* EO to soil, seeds, and cilantro plants. Figure 4 shows the EO′s effect on *E. coli* and *S.* Enteritidis in these samples. *E. coli* counts declined from 2 h postapplication, with no cells detected in soil at 60 days (Figure 4a). Simultaneously, treatment with *S. officinalis* EO consistently reduced the population of *S.* Enteritidis to 60 days, and finally, no viable cells were observed in inoculated soil at harvest (Figure 5a). Additionally, *S. officinalis* EO treatment reduced the *E. coli* population by about 1 Log_10_ CFU/g after 2 h, with further decreases until no viable cells were detected in inoculated seeds at harvest (Figure 4b). Similarly, EO treatment continually decreased the *S.* Enteritidis population, with no viable cells found in inoculated seeds at 60 days (Figure 5b). Moreover, *E. coli* counts were 4 Log_10_ CFU/g at 7 days postapplication; notably, no growth was detected at 15 and 60 days in inoculated cilantro plants (Figure 4c). Likewise, following EO application, *S.* Enteritidis count was 6.08 Log_10_ CFU/g at 2 h, but no viable cells were observed at 15 days, with complete inhibition in cilantro plants at 60 days (Figure 5c).

### 3.3. *E. coli* and *S.* Enteritidis Populations Reduction With *S. officinalis* EO

Table 3 summarizes the reductions of *E. coli* and *S.* Enteritidis populations after the application of *S. officinalis* EO. The most effective antimicrobial effect was observed in *S.* Enteritidis, with a reduction of 7.59 Log_10_ CFU/g in inoculated cilantro plants at 15 days; this result showed no significant difference compared with the reduction in inoculated soil (7.44 Log_10_ CFU/g) and seeds (7.25 Log_10_ CFU/g) at 60 days. In contrast, for *E. coli*, the highest reduction of 7.5 Log_10_ CFU/g was also observed in inoculated cilantro plants at 15 days, and this reduction was significantly greater than that observed with the other inoculated materials. Nevertheless, no significant differences were observed between inoculated soil (6.5 Log_10_ CFU/g) and seeds (5.15 Log_10_ CFU/g) with *E. coli* at 60 days. Overall, these results demonstrate the effective antimicrobial activity of *S. officinalis* EO, with reductions exceeding 7 Log_10_ CFU/g in soil, seeds, and cilantro plants.

**Table 3:** Bacterial reductions with *S. officinalis* essential oil.

Inoculated material	Persistence time	Population reduction (Log_10_ CFU/g) of *E. coli*	Population reduction (Log_10_ CFU/g) of *S.* Enteritidis
Soil	2 h	1.02 ± 0.09 CDE, d	1.48 ± 0.01 D, b
	3 days	2.01 ± 0.05 CD, bc	1.41 ± 0.03 DE, b
	7 days	2.39 ± 0.03 C, b	0.96 ± 0.08 DEF, bc
	15 days	1.50 ± 0.01 CDEF, cd	0.35 ± 0.01 EF, c
	60 days	6.50 ± 0.07 B, a	7.44 ± 0.17 A, a
Seeds	2 h	1.54 ± 0.06 CDE, b	2.78 ± 0.02 C, c
	3 days	0.53 ± 0.01 F, c	0.63 ± 0.04 DEF, d
	7 days	0.67 ± 0.01 EF, bc	4.71 ± 0.05 B, b
	15 days	0.35 ± 0.02 F, c	0.13 ± 0.01 F, f
	60 days	5.15 ± 0.15 B, a	7.25 ± 0.14 A, a
Cilantro plants	2 h	1.87 ± 0.03 CD, c	1.16 ± 0.03 CDE, c
	3 days	1.80 ± 0.08 CD, c	0.83 ± 0.01 DEF, c
	7 days	5.61 ± 0.07 B, b	4.20 ± 0.35 B, b
	15 days	7.50 ± 0.19 A, a	7.59 ± 0.09 A, a
	60 days	NG	NG

*Note:* Different capital letters indicate significant differences between the mean values of bacterial growth within each column, whereas different lowercase letters indicate significant differences within each inoculated material (Tukey′s test, *α* = 0.05; *n* = 8). Initial population 7 Log_10_ CFU/g for both bacteria. No bacterial growth was detected in negative controls (soil, seeds, and cilantro plants not inoculated).

Abbreviation: NG, no growth.

## 4. Discussion

Contamination during cilantro growth can occur due to several factors, including the use of untreated soil or inadequately composted manure, contaminated irrigation water, and exposure to wildlife or domestic animals, often associated with deficient agricultural and harvesting practices [34]. Once *E. coli* and *S*. Enteritidis are present in the soil, pathogens may persist, acting as reservoirs that facilitate the transfer of microorganisms to crops through direct contact or irrigation water. In particular, the presence of *E. coli* in soil indicates fecal contamination, which may originate from animal manure, direct livestock deposition, or even human feces. This bacterium can adhere to soil particles or be dispersed through irrigation systems, thereby contaminating the surrounding environment [35].

The persistence of *E. coli* and *S*. Enteritidis in soil is influenced by abiotic and biotic factors, such as temperature, water content, and pH are considered decisive in pathogen survival [36] demonstrated that soil pH is critical for the permanence of microorganisms, whereas *S.* Enteritidis is known to tolerate a broad pH soil range from 4 to 9. Besides, Chauret [37] observed that *E. coli* populations remain in clay soils, which is relevant because cilantro‐producing regions in the State of Puebla are characterized by this soil texture. Van der Linden et al. [38] and Underthun et al. [35] reported that *S.* Enteritidis generally shows greater environmental survival than *E. coli* in both clay and sandy soils. Oliveira et al. [39] found that *E. coli* populations persisted at 4.43 Log_10_ CFU/g after 4 weeks in soil, providing a contamination source for leafy greens such as lettuce. Likewise, Underthun et al. [35] reported that both *E. coli* and *S.* Enteritidis could persist until 224 days in clay soil. Additional evidence confirms these trends, underscoring the risk posed by these bacteria in agricultural systems. Furthermore, cilantro is frequently cultivated in Puebla under monoculture systems, which reduce soil microbial diversity and, in turn, decrease competition among microorganisms. French et al. [40] reported that this reduction in microbial antagonism facilitates the prolonged viability of pathogenic bacteria.

Another factor contributing to the survival of *E. coli* and *S.* Enteritidis in seeds is their ability to form biofilms [41]. This mechanism enhances the risk of microbiological contamination in leafy vegetables such as cilantro, which is typically consumed fresh [38, 40, 42]. In this study, the moisture content of cilantro seeds obtained (73%) allows the germination and growth of *E. coli* and *S.* Enteritidis. The contaminated seeds represent an important reservoir of enteric pathogens and a potential source of gastrointestinal infections [43]. Comparative studies support these findings, such as Shaw et al. [36], reported that *S.* Enteritidis persisted at levels above 7 Log_10_ CFU/g in seeds, whereas *E. coli* maintained populations of around 5 Log_10_ CFU/g in several crops. These results are consistent with the present research on cilantro seeds. Likewise, Beuchat and Scouten [44] demonstrated the prolonged viability of *S*. Enteritidis (3.69 Log_10_ CFU/g) than *E. coli* (2.51 Log_10_ CFU/g) in alfalfa seeds for up to 52 weeks, whereas Cui et al. [45] mentioned that *S.* Enteritidis presented effective adhesion properties in seeds with populations of 4.08, 4.21, 1.47, and 1.35 Log_10_ CFU/g, including fenugreek, alfalfa, tomato, and lettuce, respectively.

Moreover, the FDA [34] confirmed that contaminated seed germination with *S.* Enteritidis is an important cause of foodborne outbreaks of salmonellosis, and the epidemiological data support the public health significance of these findings. This colonization is due to the ability of the enteropathogens to infiltrate in pericarp and intracellular spaces within seeds. This phenomenon is associated with negative pressure generated in the pericarp by environmental factors such as temperature and water pressure [45]. When external water pressure exceeds the internal pressure of the seed, infiltration occurs, facilitated by the hydrophobic properties of the surface of the seed during germination [46]. In addition, seed germination in contaminated soil has been identified as a critical way for pathogen dissemination. Van der Linden et al. [38] reported that *E. coli* and *S.* Enteritidis can colonize aerial parts and roots of plants. In addition, *E. coli* has been shown to persist in lettuce seeds for up to 54 weeks.

Roy et al. [47] reported that both *E. coli* and *Salmonella* could persist during cilantro production due to its ability to adapt to this crop. While Castro‐Rosas and Escartin [48] observed that the main reason for *S.* Enteritidis surviving in cilantro is its capacity for internalizing in plant tissues, maintaining populations of nearly 6 Log_10_ CFU/g for periods of up to 2 years. In contrast, Oliveira et al. [39] observed that *E. coli* populations in lettuce leaves ranged from 3 to 6 Log_10_ CFU/g but declined to undetectable levels after 21 days. Furthermore, Erickson et al. [49] reported nonviable cells in lettuce of both pathogens (*E. coli* and *Salmonella* spp.) within 9 days. These results are similar to the findings of the present study, where *S.* Enteritidis and *E. coli* were undetectable in cilantro after 15 days. However, another report that survival can change in different materials, Guo et al. [50] demonstrated that *S.* Enteritidis maintained populations higher than 4 Log_10_ CFU/g in green tomatoes for up to 45 days. This prolonged retention may be due to the pathogen′s ability to infiltrate plant tissues through contact with contaminated water or soil, thereby exacerbating contamination risks. In this study, these mechanisms may help explain the existence of *S.* Enteritidis in inoculated soil for up to 15 days, as a continuous source of contamination. The results obtained in this study are consistent with the mentioned findings, confirming the persistence of *E. coli* and *S.* Enteritidis in soil, seeds, and cilantro plants.

The efficacy of *S. officinalis* EO was evaluated on the prevalence of *S.* Enteritidis and *E. coli* in seeds, soil, and cilantro plants. Conde‐Hernández et al. [21] confirmed the antimicrobial activity of *S. officinalis* EO through analyses that reported inhibition diameters and inhibition percentages of *Enterobacter agglomerans*, *Citrobacter freundii*, *Salmonella* sp., and *E. coli*.

The antimicrobial potential of this EO has been attributed to bioactive compounds such as thujone, 1,8‐cineole, and camphor [51–53]. Although Carvalho et al. [54] identified camphor as the main component of *S. officinalis*, this compound has demonstrated notable antimicrobial activity against gram positive and gram negative bacteria. Likewise, thujone is a cyclic monoterpene detected at a concentration of 13.73%, presents significant inhibitory properties, particularly against pathogenic gram‐negative bacteria such as *E. coli*, which is capable of biofilm formation [18, 55–57]. *E. coli* and *S.* Enteritidis are pathogens commonly present in agricultural soils used for fresh vegetable growth, thereby representing a potential hazard to human health [36, 57]. In this context, although the application of EOs in agriculture has been investigated, it represents a promising area for further research [58]. As shown in Table 1, the EO of *S. officinalis* contains several bioactive constituents, including flavonoids and phenolics, which demonstrate biocidal effects by disrupting bacterial cell membrane integrity [59]. Moreover, this EO has been classified as GRAS by the FDA, supporting its safety for use as an antimicrobial agent [21]. In the present study, the EO of *S. officinalis* demonstrated efficacy in reducing populations of *E. coli* and *S.* Enteritidis in inoculated soil, seeds, and cilantro plants until harvest time (60 days). Similarly, Ardalan and Shaabani [52] reported that a 5% (v/v) application of *S. officinalis* EO completely eliminated viable *S.* Enteritidis cells within 45 min. Furthermore, the antimicrobial efficacy of *S. officinalis* EO exhibits higher antimicrobial activity compared with other aromatic plant EOs, including *Rosmarinus officinalis* and *Origanum vulgare* L. [60, 61]. Recent studies provide additional context for these findings. Gheisary et al. [62] reported synergistic antimicrobial interactions between *S. officinalis*, *Origanum vulgare*, and *Cuminum cyminum* EOs against foodborne pathogens, including pathogenic *Salmonella* and *E. coli*. Similarly, Alibi et al. [63] demonstrated that EOs from aromatic plants such as *S. officinalis* significantly inhibited multidrug‐resistant *S.* Enteritidis and reduced biofilm formation. Ezzaky et al. [64], performed a meta‐analysis and confirmed the significant in vitro antimicrobial activity of EOs derived from *Salvia* spp. against major foodborne pathogens, highlighting the influence of plant species and testing methodology on efficacy. Furthermore, Toso et al. [65] evaluated oregano and rosemary EOs under different pH conditions showed that antimicrobial activity might vary depending on environmental factors, reinforcing the importance of assessing these compounds under realistic production conditions.

The antimicrobial reductions of *E. coli* and *S.* Enteritidis observed in the present study may be associated with the mechanisms of action described for EOs, including alterations in membrane permeability, interference with biofilm formation, and induction of oxidative stress in foodborne pathogens, as previously reported for plant‐derived EOs [14–17]. Moreover, recent studies have shown that synergistic interactions among EO constituents can enhance antimicrobial efficacy under complex environmental conditions, supporting the potential application of S*. officinalis* EO in preharvest agricultural systems.

In this context, the reductions observed in the present study were within the antimicrobial range previously described for plant‐derived EOs and support the applicability of *S. officinali*s EO within a soil–seed–cilantro plant system under preharvest conditions. Although this study focused on the target pathogens *E. coli* and *S*. Enteritidis isolated from cilantro crops, *S. officinalis* EO may also exhibit antimicrobial activity against other microorganisms associated with soil, seeds, or cilantro plants. Furthermore, future studies should evaluate the application of *S. officinalis* EO in unsterilized soils inoculated with the studied pathogens to better understand its interaction with the indigenous soil microbiota and the potential effects on microbial community dynamics. Finally, *S. officinalis* EO represents a promising natural antimicrobial alternative for cilantro production, with potential applications in soil, seeds, and plants during preharvest stages to improve food safety.

## 5. Conclusion

The persistence of the pathogens at populations exceeding 4 Log_10_ (CFU/g) in soil, seeds, and cilantro plants for 60 days, indicating that *E. coli* and *S.* Enteritidis can remain viable for up to the harvest. These bacteria are significant biological hazards in the context of fresh vegetable production, potentially increasing the risk of health complications for farmers and consumers. The EO of *S. officinalis* demonstrated antimicrobial activity against *E. coli* and *S.* Enteritidis in soil, seeds, and cilantro plants, with the greatest reductions of 7.5 and 7.59 Log_10_ (CFU/g) observed in cilantro plants. *S.* Enteritidis exhibited higher sensitivity to *S. officinalis* EO with all treatments in comparison with *E. coli*, emphasizing its potential as an effective and environmentally sustainable antimicrobial agent to eliminate pathogens, ensuring food safety and safeguarding public health.

## Funding

This study was supported by Vice‐Rectorate for Research and Postgraduate Studies (VIEP) at the Benemérita Universidad Autónoma de Puebla (2023‐00302).

## Conflicts of Interest

The authors declare no conflicts of interest.

## Data Availability

The data that support the findings of this study are openly available.
